# Tetracycline-controlled transgene activation using the ROSA26-iM2-GFP knock-in mouse strain permits GFP monitoring of DOX-regulated transgene-expression

**DOI:** 10.1186/1471-213X-10-95

**Published:** 2010-09-03

**Authors:** Simone Wörtge, Leonid Eshkind, Nina Cabezas-Wallscheid, Bernard Lakaye, Jinhyun Kim, Rosario Heck, Yasmin Abassi, Mustafa Diken, Rolf Sprengel, Ernesto Bockamp

**Affiliations:** 1 Max Planck Institute for Medical Research, Jahnstrasse 29, 69120 Heidelberg, Germany; 2 Medical Center of the Johannes Gutenberg-University Mainz, Institute for Toxicology, Obere Zahlbacher Str. 67, 55131 Mainz, Germany; 3 Janelia Farm Research Campus, Howard Hughes Medical Institute, 19700 Helix Drive, Ashburn, VA 20147, USA; 4 Medical Center of the Johannes Gutenberg-University Mainz, III. Department of Internal Medicine, Division of Experimental and Translational Oncology, Obere Zahlbacher Str. 63, 55131 Mainz, Germany; 5 Institute for Molecular Medicine, University Medical Center of the Johannes Gutenberg-University Mainz, 55131 Mainz, Germany

## Abstract

**Background:**

Conditional gene activation is an efficient strategy for studying gene function in genetically modified animals. Among the presently available gene switches, the tetracycline-regulated system has attracted considerable interest because of its unique potential for reversible and adjustable gene regulation.

**Results:**

To investigate whether the ubiquitously expressed *Gt(ROSA)26Sor *locus enables uniform DOX-controlled gene expression, we inserted the improved tetracycline-regulated transcription activator iM2 together with an iM2 dependent GFP gene into the *Gt(ROSA)26Sor *locus, using gene targeting to generate ROSA26-iM2-GFP **(**R26^t1Δ^) mice. Despite the presence of ROSA26 promoter driven iM2, R26^t1Δ ^mice showed very sparse DOX-activated expression of different iM2-responsive reporter genes in the brain, mosaic expression in peripheral tissues and more prominent expression in erythroid, myeloid and lymphoid lineages, in hematopoietic stem and progenitor cells and in olfactory neurons.

**Conclusions:**

The finding that gene regulation by the DOX-activated transcriptional factor iM2 in the *Gt(ROSA)26Sor *locus has its limitations is of importance for future experimental strategies involving transgene activation from the endogenous ROSA26 promoter. Furthermore, our ROSA26-iM2 knock-in mouse model (R26^t1Δ^) represents a useful tool for implementing gene function *in vivo *especially under circumstances requiring the side-by-side comparison of gene manipulated and wild type cells. Since the ROSA26-iM2 mouse allows mosaic gene activation in peripheral tissues and haematopoietic cells, this model will be very useful for uncovering previously unknown or unsuspected phenotypes.

## Backround

To understand the detailed function of particular genes we must be able to translate analytical and database information into experimental model systems. In this respect, the laboratory mouse can be used as a higher vertebrate model that permits the application of genetic tools for the *in vivo *analysis of gene function. Since constitutive, global expression of transgenes is often associated with embryonic lethality or unwanted pleiotropic effects, tight postnatal transgene regulation is the method of choice. Furthermore, correlations between phenotypes and 'ON' and 'OFF' states of the regulated gene can provide reliable cues for understanding gene-function. However, to ensure that the observed phenotypes are correlated with the gene function under investigation the reservoir of genetic tools has to be carefully evaluated.

Over the last decade the tetracycline (tet)-regulatory system has been extremely useful for generating conditional transgenic mouse models (for reviews see [1-3]). Besides its fundamental appeal as an established reversible regulatory system in mice, tet 'ON/OFF'-systems have attracted considerable interest because of their unique potential for reversible gene regulation, with the additional advantage of allowing adjustment of transgene expression levels through the administration of predetermined amounts of the pharmacological inducer doxycycline (DOX) [4-6]. The graded response of tet-regulated transgenes thus provides the unique opportunity to analyze gene dosage effects in living animals.

Although a large collection of tet-controlled transgenic mice suitable for studying gene function in many different cell types is available to the scientific community (http://www.zmg.uni-mainz.de/tetmouse for an electronically searchable database), no comprehensive analysis of a tet-regulatory effector line using the ubiquitously expressed *Gt(ROSA)26Sor *gene locus has been published. Therefore, we generated a tet 'ON' knock-in mouse line (R26^t1Δ^) containing the iM2 reverse DOX-dependent transactivator coding region [7] and an iM2 responsive GFP gene at the *Gt(ROSA)26Sor *gene locus. Analysis of R26^t1 ^mice using different iM2-responsive reporter strains revealed mosaic induction of conditional reporter gene activity in peripheral organs and in hematopoietic cells. No substantial iM2 reporter gene activation was detected in most parts of the adult brain with the exception of the *bulbus olfactorius *and, to a lesser extent, the striatum.

## Results

### Generation of the ROSA26-iM2PtetGFP knock-in mouse

**Figure 1:**
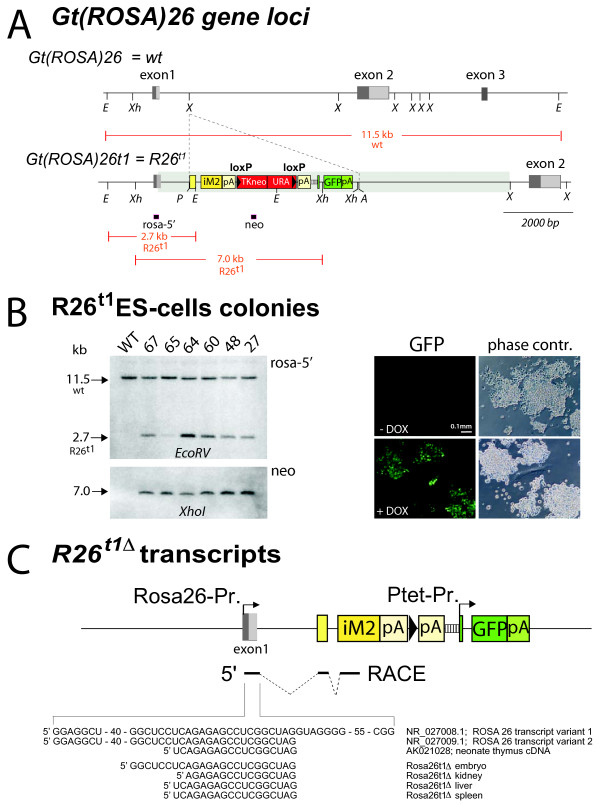
**The gene targeted *R26^t1Δ ^*allele controls the expression of DOX-inducible transcription from the endogenous promoter of the *Gt(ROSA)26Sor *locus**. **A) ***Gt(ROSA)26Sor *and *Gt(ROSA)26t1 *(*R26R^t1^*) loci. Exons are indicated as grey boxes; light grey show alternative spliced exons. Genetic elements inserted at the first *Xba*I site in intron1 of *Gt(ROSA)*26Sor are given in coloured boxes*: *first (yellow), the adenovirus major late splice acceptor sequence followed by the small intron of the adenoviral tripartite leader and the iM2 coding region terminated by the human growth hormone polyadenylation signal (hgh_pA); second (red), a loxP site (black triangles) flanked neomycin/uracil selection cassette [36] terminated by hgh_pA; third (green), the PtetO5-GFP gene module terminated by SV40_pA. Diagnostic restriction sites are indicated (E = *Eco*RV, Xh = *Xho*I, X = *Xba*I, P = *Pac*I, A = *Asc*I). The transparent green bar represents the chromosomal region covered by the targeting vector. Red-framed black boxes below *R26R^t1 ^*mark positions of Southern blot probes that detect restriction fragments given as red lines in kilo bases (kb). **B) **Left: Southern blots of genomic DNA isolated from wild type (WT) and PCR-preselected ES cell clones 27, 48, 60, 64, 65 and 67. Right: Images of R26^t1 ^ES cells show DOX-dependent GFP expression. **C) **Schematic representation of the *R26R^t1Δ ^*allele after Cre mediated removal of neo/ura at the *R26^t1 ^*allele (symbols are as in 1A). Transcriptional starts of the ROSA26 and Ptet promoter are indicated by arrows. Below the 5'-RACE identified exons (solid lines) and introns (dashed lines) of the iM2 encoding *R26^t1Δ ^*mRNA are given. Below the 5' ends of mRNA from embryos, kidney liver and spleen are aligned to *Gt(ROSA)26Sor *transcript variants of the database (NCBI Accession numbers are indicated).

By gene targeting, we inserted the iM2 transactivator gene [7], a loxP flanked neo/ura selection marker and a Ptet-controlled GFP followed by a human growth hormone polyadenylation signal into the *Xba*I site between exon1 and 2 of the *Gt(ROSA)26Sor *locus ([8]; Figure 1A). A splice acceptor sequence (SA) identical to the one used in the original *lacZ*-gene-trap allele [9] was inserted 5'-prime to the iM2 start codon to facilitate the processing of ROSA26 promoter controlled transcripts [10]. A synthetic intron in the 5'UTR of GFP was used to stabilize the Ptet transcript and an additional human growth hormone polyadenylation sequence was inserted in front of Ptet to avoid transcriptional interference between the ROSA26 and Ptet promoter. The recombinant gene locus *Gt(ROSA)26t1 *(*R26^t1^) *(Figure 1A) is thus expected to express the iM2 transactivator from the endogenous ROSA26 promoter. In the presence of DOX, iM2 can activate the Ptet-promoter, which in turn can be visualized by the expression of GFP. Thus, co-localisation of both iM2 and the Ptet responder GFP genes in the same locus facilitates a rapid fluorescent screen for monitoring the Ptet-activity in single cells.

The generation of the *R26^t1 ^*gene locus by homologous recombination in the genome of ES cells was pre-screened by PCR and confirmed by Southern blotting (Figure 1B). Six independent ES cell clones with insertion of the inducible iM2-PtetGFP module at the *Gt(ROSA)26Sor *locus were identified (Figure 1B). All six ES cell clones showed DOX-inducible GFP fluorescence (Figure 1B) and ES cell clone 64 was used for blastocyst injections to generate the ROSA26-iM2-PtetGFP mouse line, designated R26^t1^. Removal of the loxP flanked neo/ura selection cassette was performed by *in vivo *excision using the *deleter*-cre strain [11] thus generating the *R26^t1Δ ^*allele in a sub-line (R26^t1Δ^) lacking the neo/ura genes (Figure 1C).

In embryos, spleen, kidney and liver of R26^t1Δ ^mice rapid amplification of cDNA ends (RACE) revealed that *R26^t1Δ ^*transcripts contained 3'-exon1 sequences of ROSA26 transcript variant 2 (Figure 1C). Since the ROSA26 promoter and exon1 of splice variant 2 were not present in the targeting vector, this finding confirms that the recombined *R26^t1Δ ^*gene locus drives iM2 expression from the ROSA26 promoter. Thus the presence of splice variant exon1 at the 5'-end of iM2 transcripts provides additional proof for successful iM2 gene module insertion into the *Gt(ROSA)26Sor *gene locus. Currently, we cannot resolve why the exon1 splice variant 1 was not detected in our analysis. Because sequence alteration at the exon1 splice donor site could be excluded by sequencing it is possible that the presence of a second artificial intron in front of the iM2 coding sequence might influence the maturation of the primary R26^t1Δ ^transcript.

### The presence of the neo spacer sequence and the gene dosage effect on the DOX regulated GFP in the ROSA26-iM2-GFP locus

**Figure 2:**
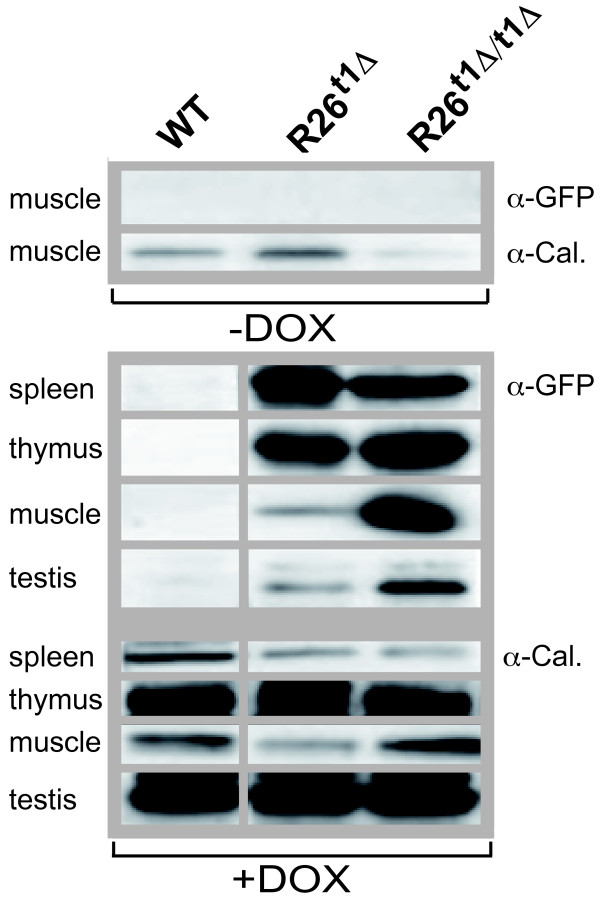
**Expression of the GFP reporter gene in adult R26t^1Δ ^and homozygous R26^t1Δ/t1Δ ^mice**. Western blot analysis of muscle extracts from wild-type (WT), R26^t1Δ ^and R26^t1Δ/t1Δ ^mice three to five month of age. Upper part: No GFP-specific signal could be detected in muscle without DOX administration. The very same blot was probed for calnexin (Cal.) and the result is shown below the GFP-specific signal. Lower part: Western blot analysis of GFP (four upper lanes) and calnexin (four lower lanes) expression in spleen, thymus, muscle and testis extracts from DOX-induced wild-type (WT), heterozygous (R26^t1Δ^) and homozygous (R26^t1Δ/t1Δ^) mice. The GFP and calnexin signals were obtained from the same blot. For reasons of simplicity the calnexin loading control and the GFP signal are aligned and pictured as one group. Animals were treated with DOX for 3 weeks prior analysis.

The iM2 activity in R26^t1Δ ^and R26^t1Δ/t1Δ ^mice was determined by GFP-detecting immunoblots of total protein extracts obtained from different tissues (Figure 2). Calnexin was used as loading control and found to be differentially expressed in various tissues. As shown in Figure 2, in the absence of DOX no GFP-specific signal was detected in any tissue of all three genotypes as exemplified for muscle tissue of R26^t1Δ ^and R26^t1Δ/t1Δ ^mice. This directly indicates that the Ptet promoter is not activated after insertion into the *Gt(ROSA)26Sor *locus by read through transcripts or promoter interference. When mice were exposed to DOX, we detected weak GFP signals only in thymus extracts from R26^t1 ^mice (not shown). However, in R26^t1Δ ^mice highest GFP levels were obtained following DOX induction in spleen, thymus, muscle and testis (Figure 2). Thus - as noticed earlier and described in detail for the *Gt(ROSA)26Sor *gene locus in ES cells [12] - removal of the NEO gene improved gene expression at the targeted locus. In homozygous R26^t1Δ ^^/t1Δ ^mice GFP expression was even further augmented in muscle and testis but this increase was not obvious in spleen and thymus. However, since spleen and thymus represent tissues with high GFP expression in activated R26^t1Δ ^animals, a two-fold increase in R26^t1Δ/t1Δ ^mice might have escaped our semi-quantitative analysis. Thus it seems that for tissues with low levels of iM2 controlled genes a gene dosage increase of iM2 and the Ptet gene(s) or both can be beneficial for the expression level of Ptet controlled genes.

### Characterisation of the R26^t1Δ ^effector mouse in hematopoietic tissues

**Figure 3:**
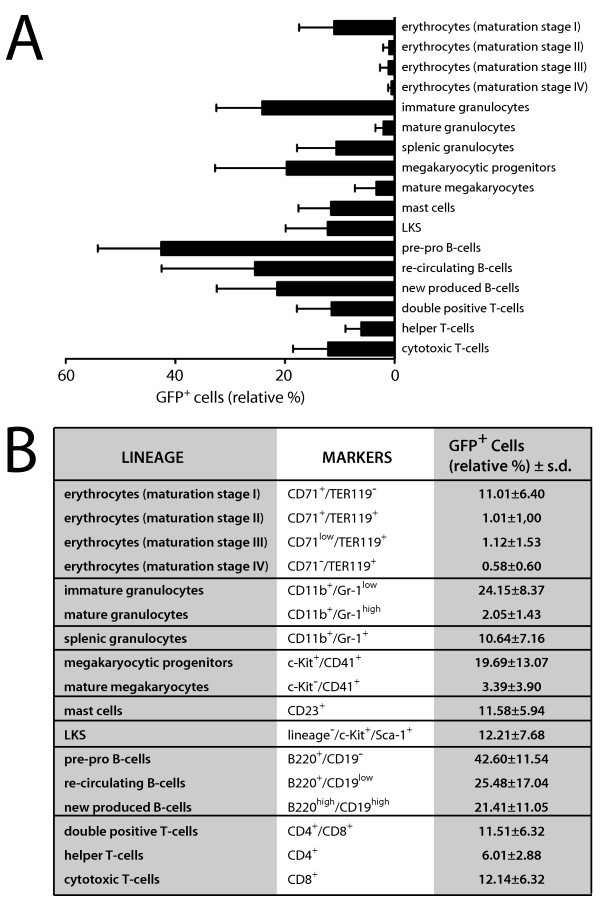
**The R26^t1Δ ^mouse line directs transgene expression to adult blood cells and hematopoietic stem and progenitor cells**. **(A) **Expression of GFP in different hematopoietic cells is given as mean values ± standard deviations in a bar graph. Analyzed cell-lineages are shown on the right. For conditional GFP activation three month old mice were exposed to DOX for fourteen days prior to the analysis. No GFP activation was detected in genetically identical littermates not exposed to DOX (not shown). At least four different animals from DOX-induced and non-induced littermate controls were analyzed for hematopoietic lineage determination. **(B) **In the right column relative percentages of GFP^+ ^cells are indicated as mean values ± standard deviations. Lineage-specific markers are specified in the central white column. Roman numerals refer to different maturation stages of red blood cell development: I, proerythroblasts; II, basophilic erythroblasts; III, late basophilic erythroblasts and chromatophilic erythroblasts; IV, orthochromatophilic erythroblasts. GFP, green fluorescent protein; LKS, lineage^-^/c-Kit^+^/Sca1^+^.

To determine to what extend different hematopoietic cell types of R26^t1Δ ^mice can implement DOX-regulated transgene expression, DOX-induced and non-induced R26^t1Δ ^mice were analyzed by flow cytometry. As expected, no GFP-expressing hematopoietic cells were found in R26^t1Δ ^mice never exposed to DOX (data not shown). In DOX-treated R26^t1Δ ^littermates GFP-expressing cells were present in all analyzed hematopoietic lineages (Figure 3). Based on their expression for CD71 and Ter119 four subpopulations representing different red blood cell maturation steps could be established (erythrocytes, maturation stage I to IV). Compared to the immature early proerythroblasts (I, 11.01% ± 6.4), GFP^+ ^cells were less abundant in more mature erythroid populations (II-IV). Analysis of bone marrow granulocytes demonstrated that about one fourth (24.15% ± 8.37) of the less mature CD11b^+^/Gr1*^low ^*population expressed GFP whereas the more differentiated CD11b^+^/Gr1*^high ^*population contained only few GFP-expressing cells (2.05% ± 1.43). In the spleen of DOX-induced R26^t1Δ ^mice 10.64% ± 7.16 of the granulocytes were GFP positive (CD11b*^+^*/Gr1*^+ ^*splenic granulocytes). Within the megakaryocytic lineage CD41^+^/c-Kit^+ ^megakaryocytic progenitors contained 19.69% ± 13.07 GFP-expressing cells while within the CD41^+^/c-Kit^- ^population of more mature megakaryocytes only 3.39% ± 3.9 of cells expressed GFP. Similarly, flow cytometric analysis of CD23^+ ^mast cells revealed the presence of a fraction of GFP-expressing cells (11.58% ± 5.94). GFP^+ ^cells were also present in the lineage negative (lin^-^), c-Kit- and Sca-1-expressing population of cells that contains hematopoietic stem cells and progenitors (12.21% ± 7.68, LKS stem cells). From the analyzed B-lymphocytes a high percentage of B220^+^/CD19^- ^pre-pro B cells (42.6% ± 11.54), re-circulating B220^+^/CD19^low ^(25.48% ± 17.04) and B220^+^/CD19^high ^new produced (21.41% ± 11.05) B-cells expressed the GFP reporter gene. Within the thymus GFP-expressing cells were found in CD4^+^/CD8^+ ^double positive cells (11.51% ± 6.32) and also in CD4 single positive (6.01% ± 2.88) and CD8 single positive (12.14% ± 6.38) thymocytes. These results indicate that the expression of the *R26^t1Δ ^*controlled iM2 transactivator can activate the co-localized Ptet-GFP transgene in erythroid, myeloid, lymphoid lineages and also in lin^-^/c-Kit^+^/Sca-1^+ ^hematopoietic stem and progenitor cells in presence of DOX. Our findings further demonstrate that the percentages of Ptet-GFP reporter gene expressing cells are highly variable in different hematopoietic cell types of R26^t1Δ^.

### Evaluation of the R26^t1Δ ^effector mouse in the brain

**Figure 4:**
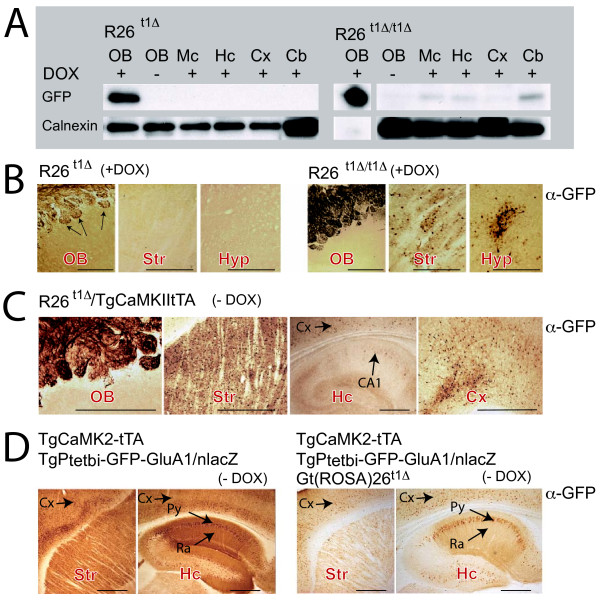
**Ptet-GFP expression of the *Gt(Rosa)26t1Δ*(*R26t^1Δ^*) locus is controlled by active iM2 and tTA in the brain of adult mice**. **(A) **Western blots for GFP and calnexin (loading control) of different brain regions in heterozygous (left, R26^t1Δ^) and homozygous (right, R26^t1Δ/t1Δ^) mice, raised and kept in the presence of DOX. In R26^t1Δ/t1Δ ^mice the intensity of the GFP signal in the olfactory bulb augmented and was adjusted by shorter exposure time and loading less sample than for other brain tissues. For the R26^t1Δ/t1Δ ^OB lane (+DOX) a shorter exposure time is shown. **(B) **Immunohistochemical analysis of GFP expression in the OB, Str and Hyp of DOX-treated heterozygous R26^t1Δ ^(left panel) and homozygous R26^t1Δ/t1Δ ^mice (right panel). Induced animals were raised and kept in the presence of DOX. **(C) **CamKII-induced GFP immunohistochemistry in OB, Str, Cx and Hc of R26^t1Δ ^mice harbouring the additional *TgCaMK2-tTA *transgene in the absence of DOX (-DOX). **(D) **GFP immunohistochemistry showing very strong CaMK2-tTA-activated GFP-GluA1 expression in Str and Hc in mice harbouring both the *TgCaMK2-tTA *and *TgPtetbi-GFP-GluA1/nlacZ *transgenes (left). Weaker GFP-GluA1 expression was apparent in brains of DOX-untreated mice (right), when in addition to the *TgCaMK2-tTA *and *TgPtetbi-GFPGluA1*/*nLacZ *transgenes the R26^t1Δ ^allele was present. Striatum (Str), cortex (Cx), mesencephalon (Mc), cerebellum (Cb), hypothalamus (Hyp), hipocampus (Hc), stratum pyramidale (Py), stratum radiatum (Ra). Black arrows indicate GFP expression. Pictures in B - D were colour adjusted in Photoshop. The scale bars for B, C and D represent 0.5 mm.

Several *Gt(ROSA)26Sor *targeted Cre-reporter mouse lines are available and have been successfully used to monitor Cre-induced transgene activation patterns in different cell types of the mouse brain [8,10,13-15]. However, results obtained with the endogenous ROSA26 promoter used for conditional gene expression in the brain were not consistent [16-19]. To clarify the potential of the R26^t1Δ ^mice for conditional transgene expression in neural tissues, protein extracts from different brain areas of R26^t1Δ ^and R26^t1Δ/t1Δ ^mice were analyzed by Western blotting (Figure 4A). A very strong DOX-dependent GFP-specific signal was present in protein extracts from the olfactory bulb of R26^t1Δ ^mice. In contrast, no GFP signal was detected in any other brain region after DOX-induction as shown for the mesencephalon, the hippocampus, the cortex and the cerebellum (Figure 4A). In homozygous, DOX-induced R26^t1Δ/t1Δ ^mice weak GFP expression was found in cerebellar extracts and to an even lower extent in the mesenencephalon, the hippocampus and the cortex. The GFP signal in the olfactory bulb was dramatically increased and ten fold less protein was loaded for immunoblotting to obtain a comparable GFP signal for the calnexin loading control.

Immunohistochemistry of sagittal brain sections confirmed strong, DOX-dependent GFP expression in the olfactory bulb of R26^t1Δ ^and R26^t1Δ/t1Δ ^mice (Figure 4B). However, the GFP-staining was restricted to the glomeruli formed between axonal projections of olfactory receptor neurons (ORNs) and dendritic processes from mitral cells of the olfactory bulb. Since the cell bodies of mitral cells within the olfactory bulb were negative for GFP immunostaining, the GFP signal in the olfactory bulb is derived from ORNs, which have their cell bodies in the nasal cavity of the olfactory epithelium outside the brain. Hardly any GFP-positive cells were apparent in brain sections of DOX-induced R26^t1Δ ^and R26^t1Δ/t1Δ ^mice; with the exception of sparse GFP-positive cells in the striatum and hypothalamus of homozygous R26^t1Δ/t1Δ ^mice (Figure 4B) directly confirming the immunoblot results (Figure 4A). Thus, as noticed in peripheral tissues, the increased gene dosage of the R26^t1Δ/t1Δ ^allele improved the expression of GFP in the brain. Our findings therefore suggest that in heterozygous R26^t1Δ ^mice, a critical threshold of DOX-activated iM2 for transcriptional activation of Ptet-GFP is not reached in most cells of the brain.

In order to increase the level of active transactivator in the brain, we generated R26^t1Δ ^mice that express the DOX-activated transcription factor tTA in the forebrain by breeding R26^t1Δ ^and TgCamKII-tTA mice [20]. Compared to R26^t1Δ ^and R26^t1Δ/t1Δ^mice, adult R26^t1Δ^/TgCamKII-tTA animals showed many more GFP-positive cells in brain regions with strong TgCamKII-tTA expression including striatum, cortex and glomeruli of the olfactory bulb thus favouring the hypothesis that detectable brain-specific GFP expression needs a certain threshold of DOX-induced activator molecules (Figure 4C). However, only a few pyramidal cells in hippocamal layer CA1 demonstrated GFP expression (Figure 4C) despite the high activity of tTA in those brain regions [20,21]. This lack of optimal tTA mediated Ptet-GFP gene activation in the *R26^t1Δ ^*locus might be caused by a negative interference of the CamKII promoter expressed tTA and R26^t1Δ ^encoded iM2 forming inactive heterodimers. The formation of inactive heterodimers is supported by mice expressing iM2 and tTA together with a Ptet-GFP responder transgene *TgPtetbi-GFP-GluA1*/*nlacZ *[21]. As described by Mack et al., TgCamKII-tTA/TgPtetbi-GFP-GluA1/*nlacZ *mice show high numbers of GFP positive cells in the forebrain (Figure 4D, left). However, in littermate animals in addition harbouring the *R26^t1Δ ^*allele, the GFP signal dramatically dropped (Figure 4D, right) demonstrating reduced activity of the tTA transactivator in presence of the *R26^t1Δ ^*allele. In summary, our results indicate that a certain threshold level of iM2 expression is needed to activate Ptet promoters and provide direct evidence that the observed lack of Ptet-mediated GFP activation in DOX induced *R26^t1Δ ^*mice might not just be the result of poor penetration of DOX through the blood brain barrier.

### Activation of R26^t1Δ ^controlled expression of Ptet regulated transgenes is DOX-dependent and inhomogeneous

**Figure 5:**
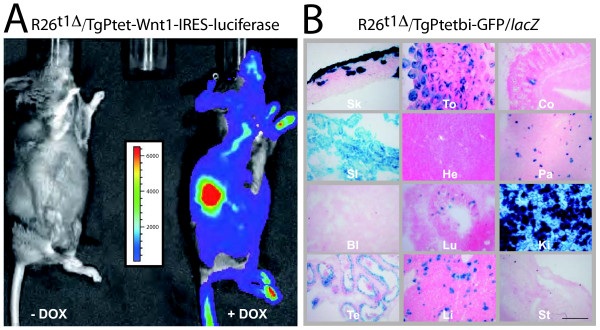
**Conditional reporter gene activation with the R26^t1Δ ^effector mouse reveals no background activity and varies in peripheral tissues of adult mice**. **(A) **Whole-body bioluminescence images of compound R26^t1Δ^/TgPtet-Wnt1-IRES-luciferase mice showing activation of luciferase after DOX exposure (right mouse, +DOX). No luciferase signal was detected in genetically identical littermates not exposed to DOX (left mouse, -DOX). The scale shown measured photon counts translated into pseudo-colours; red indicating high and blue low luciferase activity. For luciferase gene activation animals were treated for three days with 3 mg DOX/ml in the drinking water. **(B) **X-Gal staining of representative cryostat sections from different organs of induced double transgenic R26^t1Δ^/TgPtetbi-GFP/*lacZ *mice. To conditionally activate transgene expression, compound animals were exposed to 3 mg DOX/ml drinking water for two weeks. The indicated organs were dissected and analysed by X-Gal staining. Skin (Sk), tongue (To), colon (Co), small intestine (SI), heart (He), pancreas (Pa), bladder (Bl), lung (Lu), kidney (Ki), testis (Te), liver (Li), stomach (St). The scale bar represents 200 μm.

To further evaluate the functional capability of iM2 in R26^t1Δ ^mice, we recruited two different Ptet-transgenic mouse lines. First, a transgenic mouse line co-expressing Ptet-controlled Wnt1 and luciferase (TgPtet-Wnt1-IRES-luciferase) [22] to detect very low levels of transgene induction by non-invasive bioluminescence imaging [23]; second, a Ptetbi-controlled GFP/*lacZ *line for cellular resolution of iM2 activity (TgPtetbi-GFP/*lacZ*) (Figure 5).

Compound R26^t1Δ^/TgPtet-Wnt1-IRES-luciferase mice were either treated with DOX in the drinking water or were never exposed to DOX and after luciferin injection bioluminescence levels were recorded (Figure 5A). DOX-exposure during three days induced significant levels of luminescence whereas no bioluminescence signal was monitored in mice never exposed to DOX. Consistent with the previously reported variable levels of transgene expression from the *Gt(ROSA)26Sor *locus in different adult mouse tissues [16,17,24], the luminescence was not uniformly distributed in DOX-induced R26^t1Δ^/TgPtet-Wnt1-IRES-luciferase mice. This inhomogeneous luciferase activity pattern thus confirms our immunoblot observation that the *R26^t1Δ ^*locus leads to highly variable Ptet-transgene expression.

Next, *R26^t1Δ ^*induced Ptet-gene activation was visualized on a cellular level in compound R26^t1Δ^/TgPtetbi-GFP/*lacZ *mice by X-Gal staining (Figure 5B). As expected, no β-galactosidase (β-gal) expressing cells were detected without DOX treatment (data not shown). In line with the DOX-dependent induction of luciferase expression (Figure 5A), compound R26^t1Δ^/TgPtetbi-GFP/*lacZ *animals treated for two weeks with DOX showed *lacZ *reporter gene activity in skin, tongue, colon, small intestine, pancreas, lung, kidney, testis and liver. Interestingly, β-gal expression in these organs was restricted to subsets of cells. No β-gal^+ ^cells were detected in heart, bladder and stomach (Figure 5B).

Taken together our results indicated that the expression of conditional Ptet transgenes can be tightly regulated with the *R26^t1Δ ^*allele and that the *R26^t1Δ ^*mouse model implements mosaic Ptet-transgene activation in a number of peripheral tissues.

## Discussion

The promoter of the *Gt(ROSA)26Sor *locus is frequently used for ubiquitous expression of reporter genes in the mouse. These animal models represent valuable tools for conducting conditional gene expression studies. However, it is debated whether the endogenous promoter of the *Gt(ROSA)26Sor *locus can direct conditional transgene activation to all tissues of adult mice [16-19].

Now we provide additional information by a qualitative and quantitative analysis of a gene targeted mouse line (R26^t1Δ^) that contains the DOX-inducible iM2 transactivator together with an iM2-dependent GFP gene inserted into the *Gt(ROSA)26Sor *locus. We could show that after gene targeting the ROSA26 promoter drives expression of the inserted transcriptional activator gene in several tissues of R26^t1Δ ^mice. In embryo, liver, spleen and kidney exon1 of the ROSA26 transcript variant 2 was used in iM2 transcripts. If and to what extent exon1 of transcript variant 1 is used in iM2 transcripts cannot be concluded from our data, since the conducted RACE analysis did not provide a quantitative analysis of all transcripts of the ROSA26 promoter in R26^t1Δ ^mice. More importantly, exon1 transcript variant 2 is located outside of our targeting vector providing additional evidence that the *Gt(ROSA)26Sor *targeting was successful. Since electroporation with our targeting vector provided several randomly inserted GFP expressing ES cell clones, all containing transcriptional start sites in the short arm of the targeting vector (data not shown), the transcript analysis conducted here provides direct experimental proof for the ROSA26 promoter-specific transcription of the iM2 transgene and can be used as a functional test for demonstrating correct gene transcription of the rearranged locus.

In gene targeted mice ROSA26 promoter controlled iM2 activity was evaluated by monitoring (i) the co-inserted downstream Ptet-GFP reporter gene, (ii) the conditional induction of the TgPtet-Wnt1-IRES-luciferase [22] or the TgPtetbi-GFP/*lacZ *[25] transgenes. All three different reporter systems, including the very sensitive luciferase reporter, demonstrated convincingly that the *R26^t1Δ ^*allele permits gene induction with no background activity of the iM2-controlled gene in the non-induced state. These results indicate that illegitimate activation of Ptet-GFP in the *Gt(ROSA)*26Sor locus by read through transcripts or promoter interference does not take place. Our experiments also demonstrate that different iM2 responder genes could be induced by DOX. However, the responder gene expression was moderate and highly variable between and within different tissues. We detected conditional transgene expression in a subset of cells from skin, tongue, colon, small intestine, pancreas, lung, kidney, testis and liver, whereas some organs like heart, bladder and stomach had no detectable transgene induction.

In addition to the localization of conditionally activated cells within peripheral organs, we provide a detailed picture of *R26^t1Δ ^*encoded iM2 expression in the hematopoietic system. Although for the initially gene-trapped β-Geo ROSA26 mouse strain the expression of the integrated β-Geo reporter gene in immature red blood cells, lymphoid and myeloid lineages was found [24] and several ROSA26-driven reporter mouse strains exist and have been used in hematopoietic tissues [8,10,13-15], no detailed information about the potential and tissue-specificity of ROSA26-driven tet 'ON/OFF' mouse systems is available for hematopoietic tissues. To provide this missing information, we analyzed induced R26^t1Δ ^mice by flow cytometry. The results of these experiments indicate that the *R26^t1Δ ^*allele is active in different adult blood cell types and also in the lineage negative c-Kit- and Sca-1-expressing (LKS) population, which contains hematopoietic stem cells and progenitors.

In contrast to the peripheral tissues and the hematopoietic system, in cells of the central nervous system the activity of the R26^t1Δ ^encoded iM2 was barely detectable. Olfactory receptor neurons projecting to glomeroli of the olfactory bulb showed the highest DOX-inducible iM2 activity in homozygous R26^t1Δ/t1Δ ^and heterozygous R26^t1Δ ^mice. In whole brain extracts, however, only low GFP protein levels could be detected in mesencephalon, hippocampus and cortex of DOX-induced homozygous R26^t1Δ/t1Δ ^mice. Immunohistochemical staining of brain sections revealed a few, scattered GFP-expressing cells. The low levels of functional iM2 appeared to be one reason for the dysfunction of the R26^t1Δ ^allele in most cells of the central nervous system since an increase of the R26^t1Δ ^gene dosage in homozygous mice provided higher GFP expression levels in several brain regions including the glomeroli of the olfactory bulb. Even more neurons showed R26^t1Δ^-derived GFP expression when the levels of functional transactivator were increased by forebrain specific tTA expression using a CaMKII promoter driven transgene [20]. However, GFP expression was lower than in other transgenic Ptet-GFP responder mice, most likely due to the presence of the iM2 in R26^t1Δ ^animals. The reduced tTA activity in R26^t1Δ ^genotypes therefore is best explained by the mutual interference of tTA and rtTA (iM2) heterodimers [26,27] and can be convincingly visualized by the reduction of GFP-GluA1 expression in the CA1 pyramidal neurons of the hippocampus in compound transgenic mice.

Applying a very similar targeting strategy, Bäckman and colleagues recently generated a ROSA26-rtTA knock-in mouse containing a Ptet-Cre responder element inserted downstream of a ROSA26-driven rtTA cassette. Interestingly, DOX-induced adult mice expressed low Cre mRNA levels and therefore failed to activate recombination of a floxed reporter gene suggesting that the endogenous ROSA26 promoter might be too weak for efficiently inducing conditional transgene activation [28]. Besides the low activity of the endogenous ROSA26 promoter, a second reason for the poor iM2 activity in the brain might be the blood brain barrier, which may limit the free accessibility of DOX for neurons in the brain. However, using intra-cerebral DOX injection or rAAV virus mediated tTA gene delivery into the brains of adult R26^t1Δ ^mice, we failed to achieve neuronal GFP expression in the injected cortex or hippocampal areas (data not shown). Thus as described in previous studies [7] the Ptet promoter might be subject to epigenetic gene silencing when not activated during early stages of development. In this respect it is of note that in all experiments studying the expression of the *R26^t1Δ ^*GFP allele in the brain, we already applied DOX to the embryo. Similarly, we used tTA expression to activate the *R26^t1Δ ^*encoded GFP in the forebrain since the CamKII promoter of Tg-CamKII-tTA is transcriptionally active in the mouse brain during early development [25,29] and thus the Ptet promoter region is still open for transcriptional activation.

Currently, we can not formally exclude the possibility that, in addition to other effects, the lack of detectable Ptet activation in the brain might in part be caused by transcriptional interference, which is known to reduce or extinguish transcriptional activity of downstream promoters in double gene constructs [30-32] and was recently described for the CMV and the ROSA26 promoter in a targeted *Gt(ROSA)*26Sor locus in ES cells [12]. However, the fact that the R26^t1Δ^-iM2 activation of transgenic Ptet-responders in trans was limited as well, and that in the SK3 channel the insertion of a very similar linked transactivator and responder gene was operative [33] strongly argues against promoter interference. In addition, negative expression variation effects of Ptet caused by its 91 bp fragment from the CMV promoter can be responsible for the inhomogeneous activity of the Ptet promoter.

## Conclusion

The presented qualitative and semi-quantitative analysis of our R26^t1Δ ^mouse line provides detailed data on the iM2 and Ptet controlled GFP activity, which is a key information for future experiments using R26^t1Δ ^mice. This knowledge can be useful to guide the experimental design of particular research projects using DOX-regulated gene expression. Finally, the here conducted analysis provides valuable information about the potential of the R26^t1Δ ^mouse for activating transgene expression in different tissues and hematopoietic lineages and thus will help to decide if this mouse model is suitable for a particular *in vivo *experiment.

## Methods

### Construction of the targeting vector

For gene targeting at the *Gt(ROSA)26Sor *locus we inserted the coding sequence for the iM2 transactivator (rtTA-M2;[7]), containing a codon improved version of the tet-inducible M2 transcription factor [34], a loxP flanked neo selection marker and a Ptet-controlled GFP followed by a hgh polyadenylation signal into the *Pac*I/*Asc*I site between exon1 and 2 targeting vector pROSA26PA [8] to generate plasmid pROSA-iM2. In detail: a plasmid containing the tri-TAUBi-AF cassette was used as starting material [35,36]. The unique *Kpn*I and *Cla*I sites were removed from phM2-1 by filling in with Klenow polymerase followed by ligation. A 120 bp fragment containing the adenovirus major late transcription splice acceptor sequence (SA) from the intron1/exon2 boundary was amplified by PCR from pSAßGal [9] using primer SA-F (5'-TTTGGCCATACTGGCCTTAATTAATAGGGCGCAGTAGT CCAGGG-3') and SA-R (5'-TTTACTAGTACTGGAAAGACCGCGAAGAGTT-3') to introduce a *Pac*I site upstream of the SA and was inserted into pCR4 (Invitrogen, Karlsruhe, Germany) by TOPO cloning. The SA fragment was recovered by *Sfi*I and *Spe*I digestion and inserted 5' relative to the adenoviral tripartite leader sequence [37] to yield phM2-2. Subsequently, a fragment encompassing the SV40 splice donor-splice acceptor intron together with the N-terminal part of the humanized GFP (GFP [38]) expression unit [25] was amplified by PCR using primers GFP1F (5'-AAGCGCGCAAGCTTATCGATACCGTCGACC-3') and GFP1R (5'-GTATTCCAGCTTG TGGCCGAG-3'). A second fragment including the C-terminal portion of GFP and the SV40 polyadenylation signal was amplified by PCR using primer GFP2F (5'-CTCGGCCACAAGCTGGAATAC-3') and GFP2R (5'-AAGGCCGGCCGGCGCGCCCGT AATACGACTCACTATAGGG-3') to introduce an *Asc*I site between the SV40 polyadenylation signal and the *Fse*I site. Both PCR fragments were inserted into pCR4 by TOPO cloning. These two fragments, were excised using *Bss*HI/*Pfl*MI and *Pfl*MI/*Fse*I digestion respectively and ligated into the *Ase*I/*Fse*I sites of phM2-2 to place the GFP expression unit under the control of the tetracycline-responsive promoter resulting in the phM2-3 plasmid. This plasmid was then digested using *Pac*I and *Asc*I and cloned into *Pac*I and *Asc*I linearized pROSA-PA (kindly provided by S. Srinivas, Department of Physiology, Anatomy and Genetics, Oxford, UK). Plasmid pROSA-PA is a derivative of pROSA26.1 [10] where the unique *Xba*I restriction site, between exon1 and exon2 is replaced by a *Pac*I/*Swa*I/*Asc*I polylinker [8]. The resulting pROSA-hM2 plasmid was linearized with *Kpn*I and subsequently used for electroporation.

### Generation of R26^t1 ^and R26^t1Δ ^knock-in mice

The R1 embryonic stem (ES) cell line [39] was electroporated with the linearized pROSA-hM2 targeting vector and G-418-resistant clones were screened for homologous recombination by PCR with the GC-Rich PCR system (Roche, Mannheim, Germany) using primers ROSA-IN (5'-CCTAAAGAAGAGGCTGTGCTTTGG-3') and SA-IN (5'-CATCAAGGAAACCCTGGACTACTG-3'). Furthermore, homologous recombination of ES cells was analyzed by Southern blotting using an 80 nucleotide probe [10] located mainly upstream of the 5' homology region (5' rosa probe) and an 624 nucleotide neomycin probe (neo probe). Correctly recombined ES cell clone 64 was injected into blastocysts to generate chimeric mice. Successful germ-line transmission and correct integration was confirmed by PCR, using primers ROSA-IN and SA-IN. For *in vivo *excision of the neomycin-resistant cassette, germ-line-transmitting R26^t1 ^knock-in mice were crossed to the *deleter*-cre strain [11]. Successful excision of the loxP flanked neo/ura cassette was confirmed by PCR. Genotyping was performed using a three primer PCR approach with oligonucleotides ROSA01 (5'-TTCCCTCGTGATCTGCAACTCC-3') as forward primer and oligonucleotides ROSA02 (5'-GCTTCAGATGTGCCTTGCTCTC-3') and ROSA07 (5'-CATCAGACTTCTAAGATCAGG-3') as reverse primers under standard PCR conditions, yielding products of 565 bp for the *Gt(ROSA)26Sor *allele and 916 bp for the R26^t1Δ ^allele respectively. Gene targeted mice were generated under the licence Az.: 35-9185.81/G-74/07 (Regierungspräsidium Karlsruhe, Germany).

### Animals

The transgenic mouse lines *deleter*-*Cre*, TgPtet-Wnt1-IRES-luciferase, TgCamKII-tTA, TgPtetbi-GFP/*lacZ *and the TgPtetbi-GFP-GluA1/*nlacZ *have been described previously [11,20-22,25]. To induce transgene expression animals were administered a solution of doxycycline (DOX, Sigma-Aldrich, Taufkirchen, Germany) in water containing 1% sucrose. For each experiment the exposure times and DOX concentrations used are specified. DOX treatment was performed under the licence 35.-9185.82/A49/06, Regierungspräsidium Karlsruhe. Terminal experiments under the license MPI/T-15/08 and 177-07/981-18 Landesuntersuchungsamt Rheinland-Pfalz.

### RNA isolation and RACE

Total RNA from mice tissues was homogenized using an Utra Turrax (IKA, Staufen; Germany) and RNA was isolated with TRI REAGENT^®^ (Molecular Research Center Inc. Cincinnati, OH 45212, USA) followed by phenol/chloroform purification. The 5' RACE was performed using the FirstChoice^®^ RLM-RACE kit (Ambion, Applied Biosystems, Darmstadt Germany) according to the manufacturer instructions. The 5' RLM PCR was performed using in the first reaction the itTA-race out primer (5'-TCTGTAGGCCTGGTGCCCAAGTG-3') and for the nested PCR the itTA-race in primer (5'-CCCTTCCAGAGGGCAGAAGTGGGTG-3') was used. The PCR products were directly subcloned into the pCR4-topo vector using TOPO^®^ cloning technology (Invitrogen, Darmstadt, Germany) and subsequently sequenced. Sequences were analyzed using Lasergene (DNAStar.COM; Madison, Wi, USA). Sequences for the RACE iM2 transcripts of the R26^t1Δ ^allele were annotated to the NCBI database and are available under the accession number HM748862.

### *In vivo *bioluminescence imaging

DOX-induced (3 mg DOX/ml for three days) and non-induced compound R26^t1Δ^/Ptet-Wnt1-IRES-luciferase mice were intraperitoneally injected with luciferin (150 mg/kg body weight, Becton Dickinson, Heidelberg, Germany) and anesthetized by continuous inhalation with isofluorane (Merck, Darmstadt, Germany). After waiting for five minutes to allow distribution of luciferin, the mice were placed in the chamber of an IVIS Lumina optical imaging system (Caliper Life Sciences, Rüsselsheim, Germany) and bioluminescence levels were collected for 15 seconds. The signal intensity was scaled to a pseudocolor image, which was then superimposed on a grayscale photo of the mice using Living Image software v. 3.0 (Caliper Life Sciences, Rüsselsheim, Germany).

### Southern and Western blotting

Southern blots were performed employing standard procedures using an outside ROSA26 probe as described previously [10] and as an internal neomycin-specific (neo) probe, a 624 bp DNA fragment amplified from the neo gene with the primers rspneo4 (5'-GGC TATTCGGC TATGAC TGGGC -3') and rspneo5 (5'-GGGTAGCCAACGC TATGTCC TG-3').

For Westen blot analysis proteins were isolated with an Ultra Turrax in 25 mM HEPES (ph 7,4) containing protease inhibitors (Complete, EDTA-free; Roche, Mannheim, Germany). The homogenates were centrifuged (900 g; 10 min at 4°C) to remove cell debris and 10-25 μg of protein was resolved on a 8-12% SDS-polyacrylamid gel, transfered onto nitrocellulose membranes and probed with anti-GFP (Abcam; ab6556 1:5000, Abcam, Cambridge, UK) and anti-calnexin antibodies (1:3000, Stressgene, Ann Arbor, USA).

### Histology and X-Gal staining

For immunohistology mice were anesthetized with isofluoran (Abbott, Ludwigshafen, Germany) and perfused with PBS/4% paraformaldehyde (PFA, Sigma-Aldrich). The brain was isolated and post fixed in PBS/4%PFA for an additional 3 to 12 hours. For immunohistochemistry, 40- to 70-μm thick vibratom slices were used. Antibody staining on floating vibratom sections was performed as described before [40] using the primary polyclonal anti-GFP primary antibody (Abcam; ab6556 1:5000) together with the anti-rabbit antibody coupled to horseradish peroxidase (Vector Laboratories, 1:600, Vector Laboratories, Eching, Germany) as the secondary antibody. Staining was visualized with 3-3' diaminobenzidine (DAB, Fluka GmbH, Deisenhofen, Germany), mounted on slides and air-dried. DAB-developed slides were coversliped with Eukitt mounting medium (Kindler GmbH, Freiburg, Germany). Staining for X-Gal was performed as previously described [41]. All sections were counterstained with fast red (Sigma-Aldrich, Taufkirchen, Germany). Images were captured using a colour view digital camera running on an Olympus BX50 WI microscope. Images were digitalized using the analySIS software package (Soft Image Systems, Münster, Germany) and imported into Photoshop.

### Flow cytometry

Cells acquisition was performed on a FACSCalibur or LSRII cytometer (BD) and analyzed using the FlowJo software (Ashland, USA). For lineage determination cells were analyzed as previously described [41]. Before flow cytometry, blood cells from DOX-induced (14 days of 3 mg/ml DOX) or non-induced (normal drinking water) ROSA26-iM2 R26^t1Δ ^mice were pre-incubated with PBS and supplemented with 5% rat serum for 30 minutes to reduce nonspecific binding. Dead cells were excluded by 7-amino-actinomycin D (Becton Dickinson, Heidelberg, Germany) staining. For cell staining we used antibodies (all from Becton Dickinson, Heidelberg, Germany) directed against the following: CD4 (GK1.5), CD8 (53-6.7), B220 (RA3-6B2), CD19 (1D3), Gr1 (RB6-8C5), CD11b/Mac1 (M1/70), Ter119, CD71 (R17 217.1.4), CD41 (MWReg30), c-Kit (2B8), Sca-1 (D7). The CD23 antibody was from Southern Biotech, Birmingham, USA. In all cases, the number of GFP-expressing cells was determined in four independent experiments analyzing, each time, a minimum of 5 × 10^5 ^cells.

### Statistical analysis

For statistical data analysis the two-tailed *t *test was applied using the Prism4 package for Windows (GraphPad software, La Jolla, USA).

## Authors' contributions

SW carried out most of the experiments, helped to draft the manuscript. LE prepared sections and supervised and planned flow cytometric experiments. NCW made most of the FACS analysis. BL cloned the targeting construct, participated in the targeting, performed southern blot analysis, performed some of the FACS analysis. JK performed codon improvement of the iM2. RH made the cryosections and established the acetone fixations. YA performed the *lacZ *stains. MD performed the two-photon imaging analysis. RS has designed the original study, edited the manuscript. EB designed remaining experiments and wrote the manuscript. All authors have read and approved the manuscript.

## References

[B1] Bockamp E Maringer M Spangenberg C Fees S Fraser S Eshkind L Oesch F Zabel B Of mice and models: improved animal models for biomedical research Physiol Genomics 2002 11 115 32 12464688 10.1152/physiolgenomics.00067.2002

[B2] Bockamp E Sprengel R Eshkind L Lehmann T Braun JM Emmrich F Hengstler JG Conditional transgenic mouse models: from the basics to genome-wide sets of knockouts and current studies of tissue regeneration Regen Med 2008 3 217 35 10.2217/17460751.3.2.217 18307405

[B3] Sprengel R Hasan MT Tetracycline-controlled genetic switches Handb Exp Pharmacol 2007 49 72 full_text 17203651 10.1007/978-3-540-35109-2_3

[B4] Katsantoni EZ Anghelescu NE Rottier R Moerland M Antoniou M de Crom R Grosveld F Strouboulis J Ubiquitous expression of the rtTA2S-M2 inducible system in transgenic mice driven by the human hnRNPA2B1/CBX3 CpG island BMC Dev Biol 2007 7 108 10.1186/1471-213X-7-108 17900353 PMC2080639

[B5] Bornkamm GW Berens C Kuklik-Roos C Bechet JM Laux G Bachl J Korndoerfer M Schlee M Holzel M Malamoussi A Stringent doxycycline-dependent control of gene activities using an episomal one-vector system Nucleic Acids Res 2005 33 e137 10.1093/nar/gni137 16147984 PMC1201338

[B6] Roth S Franken P van Veelen W Blonden L Raghoebir L Beverloo B van Drunen E Kuipers EJ Rottier R Fodde R Generation of a tightly regulated doxycycline-inducible model for studying mouse intestinal biology Genesis 2009 47 7 13 10.1002/dvg.20446 18942097

[B7] Zhu P Aller MI Baron U Cambridge S Bausen M Herb J Sawinski J Cetin A Osten P Nelson ML Silencing and un-silencing of tetracycline-controlled genes in neurons PLoS One 2007 2 e533 10.1371/journal.pone.0000533 17579707 PMC1888723

[B8] Srinivas S Watanabe T Lin CS William CM Tanabe Y Jessell TM Costantini F Cre reporter strains produced by targeted insertion of EYFP and ECFP into the ROSA26 locus BMC Dev Biol 2001 1 4 10.1186/1471-213X-1-4 11299042 PMC31338

[B9] Friedrich G Soriano P Promoter traps in embryonic stem cells: a genetic screen to identify and mutate developmental genes in mice Genes Dev 1991 5 1513 23 10.1101/gad.5.9.1513 1653172

[B10] Soriano P Generalized lacZ expression with the ROSA26 Cre reporter strain Nat Genet 1999 21 70 1 10.1038/5007 9916792

[B11] Schwenk F Baron U Rajewsky K A cre-transgenic mouse strain for the ubiquitous deletion of loxP-flanked gene segments including deletion in germ cells Nucleic Acids Res 1995 23 5080 1 10.1093/nar/23.24.5080 8559668 PMC307516

[B12] Strathdee D Ibbotson H Grant S Expression of transgenes targeted to the Gt(ROSA)26Sor locus is orientation dependent PLoS One 2006 1 e4 10.1371/journal.pone.0000004 17183668 PMC1762389

[B13] Mao X Fujiwara Y Chapdelaine A Yang H Orkin SH Activation of EGFP expression by Cre-mediated excision in a new ROSA26 reporter mouse strain Blood 2001 97 324 6 10.1182/blood.V97.1.324 11133778

[B14] Luche H Weber O Nageswara Rao T Blum C Fehling HJ Faithful activation of an extra-bright red fluorescent protein in "knock-in" Cre-reporter mice ideally suited for lineage tracing studies Eur J Immunol 2007 37 43 53 10.1002/eji.200636745 17171761

[B15] Stoller JZ Degenhardt KR Huang L Zhou DD Lu MM Epstein JA Cre reporter mouse expressing a nuclear localized fusion of GFP and beta-galactosidase reveals new derivatives of Pax3-expressing precursors Genesis 2008 46 200 4 10.1002/dvg.20384 18395835 PMC2747029

[B16] Hameyer D Loonstra A Eshkind L Schmitt S Antunes C Groen A Bindels E Jonkers J Krimpenfort P Meuwissen R Toxicity of ligand-dependent Cre recombinases and generation of a conditional Cre deleter mouse allowing mosaic recombination in peripheral tissues Physiol Genomics 2007 31 32 41 10.1152/physiolgenomics.00019.2007 17456738

[B17] Seibler J Zevnik B Kuter-Luks B Andreas S Kern H Hennek T Rode A Heimann C Faust N Kauselmann G Rapid generation of inducible mouse mutants Nucleic Acids Res 2003 31 e12 10.1093/nar/gng012 12582257 PMC150244

[B18] Hochedlinger K Yamada Y Beard C Jaenisch R Ectopic expression of Oct-4 blocks progenitor-cell differentiation and causes dysplasia in epithelial tissues Cell 2005 121 465 77 10.1016/j.cell.2005.02.018 15882627

[B19] Jullien N Goddard I Selmi-Ruby S Fina JL Cremer H Herman JP Use of ERT2-iCre-ERT2 for conditional transgenesis Genesis 2008 46 193 9 10.1002/dvg.20383 18395834

[B20] Mayford M Bach ME Huang YY Wang L Hawkins RD Kandel ER Control of memory formation through regulated expression of a CaMKII transgene Science 1996 274 1678 83 10.1126/science.274.5293.1678 8939850

[B21] Mack V Burnashev N Kaiser KM Rozov A Jensen V Hvalby O Seeburg PH Sakmann B Sprengel R Conditional restoration of hippocampal synaptic potentiation in Glur-A-deficient mice Science 2001 292 2501 4 10.1126/science.1059365 11431570

[B22] Gunther EJ Moody SE Belka GK Hahn KT Innocent N Dugan KD Cardiff RD Chodosh LA Impact of p53 loss on reversal and recurrence of conditional Wnt-induced tumorigenesis Genes Dev 2003 17 488 501 10.1101/gad.1051603 12600942 PMC195997

[B23] Contag CH Bachmann MH Advances in in vivo bioluminescence imaging of gene expression Annu Rev Biomed Eng 2002 4 235 60 10.1146/annurev.bioeng.4.111901.093336 12117758

[B24] Zambrowicz BP Imamoto A Fiering S Herzenberg LA Kerr WG Soriano P Disruption of overlapping transcripts in the ROSA beta geo 26 gene trap strain leads to widespread expression of beta-galactosidase in mouse embryos and hematopoietic cells Proc Natl Acad Sci USA 1997 94 3789 94 10.1073/pnas.94.8.3789 9108056 PMC20519

[B25] Krestel HE Mayford M Seeburg PH Sprengel R A GFP-equipped bidirectional expression module well suited for monitoring tetracycline-regulated gene expression in mouse Nucleic Acids Res 2001 29 E39 10.1093/nar/29.7.e39 11266574 PMC31300

[B26] Freundlieb S Schirra-Muller C Bujard H A tetracycline controlled activation/repression system with increased potential for gene transfer into mammalian cells J Gene Med 1999 1 4 12 10.1002/(SICI)1521-2254(199901/02)1:1<4::AID-JGM4>3.0.CO;2-Y 10738580

[B27] Rossi F Blau H Recent advances in inducible gene expression systems Curr Opin Biotechnol 1998 9 451 6 10.1016/S0958-1669(98)80028-1 9821271

[B28] Backman CM Zhang Y Malik N Shan L Hoffer BJ Westphal H Tomac AC Generalized tetracycline induced Cre recombinase expression through the ROSA26 locus of recombinant mice J Neurosci Methods 2009 176 16 23 10.1016/j.jneumeth.2008.08.024 18801387

[B29] Krestel HE Shimshek DR Jensen V Nevian T Kim J Geng Y Bast T Depaulis A Schonig K Schwenk F A genetic switch for epilepsy in adult mice J Neurosci 2004 24 10568 78 10.1523/JNEUROSCI.4579-03.2004 15548671 PMC6730297

[B30] Hasegawa K Nakatsuji N Insulators prevent transcriptional interference between two promoters in a double gene construct for transgenesis FEBS Lett 2002 520 47 52 10.1016/S0014-5793(02)02761-8 12044868

[B31] Kadesch T Berg P Effects of the position of the simian virus 40 enhancer on expression of multiple transcription units in a single plasmid Mol Cell Biol 1986 6 2593 601 3023940 10.1128/mcb.6.7.2593-2601.1986 PMC367815

[B32] Proudfoot NJ Transcriptional interference and termination between duplicated alpha-globin gene constructs suggests a novel mechanism for gene regulation Nature 1986 322 562 5 10.1038/322562a0 3736674

[B33] Bond CT Sprengel R Bissonnette JM Kaufmann WA Pribnow D Neelands T Storck T Baetscher M Jerecic J Maylie J Respiration and parturition affected by conditional overexpression of the Ca2+-activated K+ channel subunit, SK3 Science 2000 289 1942 6 10.1126/science.289.5486.1942 10988076

[B34] Urlinger S Baron U Thellmann M Hasan MT Bujard H Hillen W Exploring the sequence space for tetracycline-dependent transcriptional activators: novel mutations yield expanded range and sensitivity Proc Natl Acad Sci USA 2000 97 7963 8 10.1073/pnas.130192197 10859354 PMC16653

[B35] Jerecic J Schulze CH Jonas P Sprengel R Seeburg PH Bischofberger J Impaired NMDA receptor function in mouse olfactory bulb neurons by tetracycline-sensitive NR1 (N598R) expression Brain Res Mol Brain Res 2001 94 96 104 10.1016/S0169-328X(01)00221-2 11597769

[B36] Storck T Krüth U Kolhekar R Sprengel R Seeburg P Rapid construction in yeast of complex targeting vectors for gene manipulation in the mouse Nucleic Acids Res 1996 24 4594 6 10.1093/nar/24.22.4594 8948655 PMC146279

[B37] Logan J Shenk T Adenovirus tripartite leader sequence enhances translation of mRNAs late after infection Proc Natl Acad Sci USA 1984 81 3655 9 10.1073/pnas.81.12.3655 6587381 PMC345277

[B38] Zolotukhin S Potter M Hauswirth WW Guy J Muzyczka N A "humanized" green fluorescent protein cDNA adapted for high-level expression in mammalian cells J Virol 1996 70 4646 54 8676491 10.1128/jvi.70.7.4646-4654.1996 PMC190401

[B39] Nagy A Rossant J Nagy R Abramow-Newerly W Roder J Derivation of completely cell culture-derived mice from early-passage embryonic stem cells Proc Natl Acad Sci USA 1993 90 8424 8 10.1073/pnas.90.18.8424 8378314 PMC47369

[B40] Spergel DJ Kruth U Hanley DF Sprengel R Seeburg PH GABA- and glutamate-activated channels in green fluorescent protein-tagged gonadotropin-releasing hormone neurons in transgenic mice J Neurosci 1999 19 2037 50 10066257 10.1523/JNEUROSCI.19-06-02037.1999 PMC6782541

[B41] Bockamp E Antunes C Maringer M Heck R Presser K Beilke S Ohngemach S Alt R Cross M Sprengel R Tetracycline-controlled transgenic targeting from the SCL locus directs conditional expression to erythrocytes, megakaryocytes, granulocytes, and c-kit-expressing lineage-negative hematopoietic cells Blood 2006 108 1533 41 10.1182/blood-2005-12-012104 16675709

